# The influence of systemically or locally administered mesenchymal stem cells on tissue repair in a rat oral implantation model

**DOI:** 10.1186/s40729-017-0112-4

**Published:** 2018-01-13

**Authors:** Miya Kanazawa, Ikiru Atsuta, Yasunori Ayukawa, Takayoshi Yamaza, Ryosuke Kondo, Yuri Matsuura, Kiyoshi Koyano

**Affiliations:** 1 Section of Implant and Rehabilitative Dentistry, Division of Oral Rehabilitation, Faculty of Dental Science, Kyushu University, 3-1-1 Maidashi, Higashi-ku, Fukuoka, 812-8582 Japan; 2 Department of Molecular Cell and Oral Anatomy, Faculty of Dental Science, Kyushu University, Fukuoka, Japan

**Keywords:** Mesenchymal stem cell, Dental implant, Epithelial cell, Systemic and local administration

## Abstract

**Background:**

Multipotent mesenchymal stem cells (MSCs) are used clinically in regenerative medicine. Our previous report showed systemically injected MSCs improved peri-implant sealing and accelerated tissue healing. However, the risks of systemic MSC administration, including lung embolism, must be considered; therefore, their local application must be assessed for clinical safety and efficacy. We investigated differences in treatment effect between local and systemic MSC application using a rat oral implantation model.

**Methods:**

Rat bone marrow-derived MSCs were isolated and culture-expanded. The rat’s right maxillary first molars were extracted and replaced with experimental titanium implants. After 24 h, MSCs (1 × 10^6^/ml) were systemically or locally injected into recipient rats via the tail vein (systemic group) or buccal subcutaneous tissue (local group), respectively. Rats treated in the absence of MSCs were included as a control (control group). The maxillary epithelium was assessed histologically after 4 weeks to evaluate laminin-332 (Ln-332) distribution and horseradish peroxidase invasion, as indicators of peri-implant epithelium (PIE) formation and PIE sealing to the implant surface, respectively. The effect of MSCs on rat oral epithelial cell (OEC) morphology was determined by coculture.

**Results:**

Systemic group MSCs accumulated early at the peri-implant mucosa, while local group MSCs were observed in various organs prior to later accumulation around the implant surface. PIE formation and Ln-332-positive staining at the implant interface were enhanced in the systemic group compared with the local and control groups. Furthermore, OEC adherence on implants was reduced in high-density compared with low-density MSC cocultures.

**Conclusions:**

Local MSC injection was more ineffective than systemic MSC injection at enhancing PIE sealing around titanium implants. Thus, although local MSC administration has a wide range of applications, further investigations are needed to understand the exact cellular and molecular mechanisms of this approach prior to clinical use.

## Background

Mesenchymal stem cell (MSC)-based approaches can be broadly divided into two categories: cell therapy and regenerative medicine. Cell therapy is focused on the anti-inflammatory, immune-regulatory, and homeostasis-regulatory actions of MSCs to treat disorders like malignant lymphoma, angina pectoris, and atopic dermatitis. Conversely, regenerative medicine is focused on MSCs playing a tissue engineering role, to enhance tissue regeneration using growth factors and scaffolds; for example, to generate tissue-engineered skin or cartilage, which have been assessed in clinical trials.

Our previous study showed that systemically injected MSCs improved attachment of the peri-implant epithelium (PIE) to the titanium (Ti) implant surface and accelerated tissue healing around the implant. Because the systemically injected MSCs accumulated around the experimental implant, we believe they acted through both regenerative medicine and cell therapy modes [1]. Indeed, the peri-implant tissue is always exposed to the possibility of inflammation because the Ti implant penetrates through the oral mucosa. However, many studies have shown that the PIE has a low sealing ability within the oral environment [2–4], meaning bacteria can more readily accumulate around the implant and induce inflammatory destruction more easily than around the natural tooth [5, 6]. Additionally, it is important to prevent epithelial down-growth by promoting epithelial cell adherence and stabilizing the epithelial soft tissue seal [7]. Therefore, improving local defense within the mucosa is indispensable to enabling successful implantation.

Some studies report that epithelial healing after implant replacement is similar to mucosa wound healing [8]. Wound healing goes through a genetically programmed repair process involving inflammation, cell proliferation, re-epithelialization, formation of granulation tissue, angiogenesis, interactions between various cell types, and matrix/tissue remodeling [9]. Therefore, the aim of MSC treatment is to regulate many cells to restore the structure, function, and physiology of damaged tissues around the implant [10].

Accumulation of MSCs adjacent to the damaged tissue following their administration into an implant model can be determined following “systemic” or “local” transplantation. Although systemic MSC administration has proven efficacious and has a large advantage as our above previous studies [11, 12], possible risks, including pulmonary embolism, pose a serious issue [13, 14]. It is therefore important to provide an alternative low-risk method that avoids MSCs becoming trapped within healthy organs. Despite this, cell regulation following local cell administration is not well-documented with respect to peri-implant tissue regeneration.

The purpose of this study was to verify the effects and mechanisms of bone marrow-derived MSCs following their local administration using an oral implantation rat model, to deepen our understanding of this approach for effective utilization of MSCs.

## Methods

### MSC isolation

Bone marrow cells were flushed out of the femurs and tibias of 4-week-old green fluorescent protein-transgenic Wistar rats. Cells were treated with a 0.85% NH_4_Cl solution for 10 min to lyse the red blood cells and were passed through a 70-μm cell strainer to obtain a single cell suspension. Cells were seeded into 100-mm plastic culture dishes (1 × 10^6^ cells/dish), washed with phosphate buffered saline (PBS), and cultured in growth medium consisting of alpha minimum essential medium (α-MEM; Invitrogen, Grand Island, NY, USA), 20% fetal bovine serum (Equitech-Bio, Kerrville, TX), 2 mM L-glutamine (Invitrogen), 55 μm 2-mercaptoethanol (Invitrogen), 100 U/ml penicillin, and 100 μg/ml streptomycin (Invitrogen). Passage 3 (P3) cells were used experimentally in this study.

### Immunofluorescent staining

MSCs (5 × 10^4^ cells/ml) were seeded into 35-mm dishes and incubated for 12 h at 37 °C in 5% CO_2_. The slides were then fixed in 4% paraformaldehyde (PFA) for 5 min and blocked with secondary antibody-matched normal serum for 1 h, followed by incubation with mouse anti-rat CD44, CD90, and CD105 antibodies (1:100, Sigma-Aldrich, St. Louis, MO,) overnight at 4 °C. The slides were then treated with fluorescein isothiocyanate (FITC)-conjugated secondary antibodies (1:200, Jackson Immuno Research, West Grove, PA) for 1 h at room temperature and mounted using VECTASHIELD® Mounting Medium containing 4′6-diamidino-2-phenylindole (DAPI) (Vector Laboratories, Burlingame, CA).

### Experimental implants

**Fig. 1:**
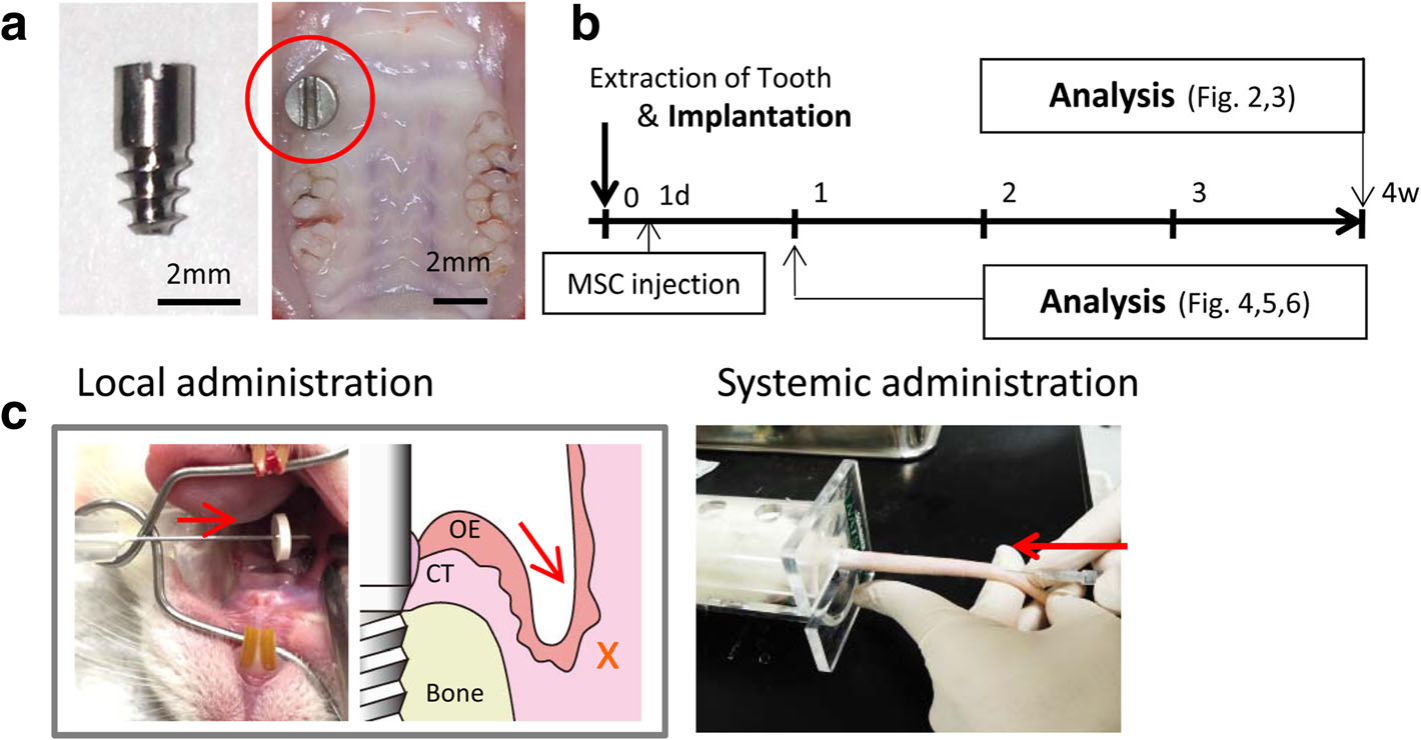
In vivo experimental design. **a** Photographs of the experimental implant (left panel) and implant in the rat oral cavity (right panel). **b** Experimental protocol of in vivo study: Implantation was performed at the same time as tooth extraction, then 1 day after implantation, mesenchymal stem cells (MSCs) were injected via the tail vein or into the gingivobuccal fold around the dental implant. Epithelial tissue structure around the implant was observed after 4 weeks. MSC accumulation into various tissues was observed 1, 3, 5, and 7 days after MSCs injection. **c** Photograph of MSC administration into the model rat with the experimental implant

Single piece, screw-type pure Ti (Japan Industrial Standards Class 1; equivalent to ASTM Grade 1) experimental implants with a machine-polished surface (Sky blue, Fukuoka, Japan) were used in accordance to our previous study [15] (Fig. 1a). Implant surface roughness was measured using a laser scanning microscope (VK-9710, Keyence, Osaka, Japan), and the arithmetic mean roughness (Ra) was found to be 0.16 μm. The implants were treated with 100% acetone and autoclave sterilized before use.

### Oral implantation

All experimental procedures were approved by the Ethics Committee on Animal Experimentation at Kyushu University (Approval Number: A25-133-0), Japan, in accordance with the ARRIVE guidelines and the Guidelines of the Japanese Physiological Society. Surgical implantation was performed in accordance with previously published protocols [4, 16]. Briefly, 6-week-old Wistar rats (27 males, 120–150 g) underwent immediate implant placement as follows: the maxillary right first molars were removed and an experimental implant was screwed into the socket under systemic chloral hydrate under systemic anesthesia.

### MSC administration

Twenty-four hours after implantation, rats were lightly anesthetized with chloral hydrate and lidocaine hydrochloride, and ex vivo expanded P3 green fluorescent protein (GFP)-MSCs (1 × 10^6^ cells) were administrated via one of the following modes: (1) systemic injection via the tail vein (systemic group), (2) local injection into the gingivobuccal fold around the dental implant (local group), and (3) no MSC injection (control group).

### Smear staining

Peripheral blood was collected from the retro-orbital plexus of the model rats. Samples were spread onto slides, dried for 30 min, and fixed in 4% PFA for 10 min. For fluorescence staining, samples were incubated with FITC-conjugated rat anti-GFP antibodies for 2 h at 37 °C. Imaging was performed using an Axiotech Microscope (Carl Zeiss, Göttingen, Germany).

### Enzyme-linked immunosorbent assay for inflammatory markers

Peripheral blood was centrifuged to obtain the blood serum, corrected 24 h after the MSC administration. The supernatants from the blood were extracted using M-PER® (Thermo Fisher Scientific, Waltham, MA) mammalian protein extraction reagent. The samples were centrifuged and used in an enzyme-linked immunosorbent assay (ELISA) for detection of interleukin (IL)-2, IL-4, and IL-10 (R&D Systems).

### Instillation of horseradish peroxidase

The procedure for topical application of horseradish peroxidase (HRP) was similar to that reported previously [16, 17]. Four weeks after implantation, rats were placed under deep anesthesia, and 50 mg/ml of HRP (type 11, molecular weight 40,000 Da, Sigma-Aldrich) was instilled into the oral mucosa surrounding the implant for 60 min. The optimal length of HRP penetration was estimated using peroxidase 3,3′-diaminobenzidine (DAB, Nacalai tesque, Kyoto, Japan) and hematoxylin staining.

### Tissue preparation and immunohistochemistry for immunofluorescence

Tissues were prepared in accordance to the methods described in our previous studies [8, 15]. After each experimental period, the rats were deeply anesthetized and perfused intracardially with heparinized PBS followed by 4% PFA (pH 7.4). Tissue samples (lung, liver, heart, and maxilla) were collected at each experimental period, dissected, and immersed in 4% PFA for 48 h at 4 °C. The oral mucosa surrounding the implant and tooth site was carefully removed from the bone, implant, or tooth, and cut into 10-μm bucco-palatal sections using a cryostat at − 20 °C. The sections were then stained immunohistochemically using mouse anti-rat GFP (1:100, Sigma-Aldrich), CD90 (1:100, Sigma-Aldrich), and Ln-332 (1:100, Santa Cruz Biotechnology, Santa Cruz, CA) antibodies (1:100, Sigma-Aldrich) overnight at 4 °C. Samples were then treated with FITC-conjugated secondary antibody (1:200, Jackson Immuno Research, West Grove, PA, USA) for 1 h at room temperature and mounted with DAPI (Vector Laboratories), as described previously [17, 18].

### Detection of cell apoptosis

For apoptosis detection, the 10-μm bucco-palatal sections from around the experimental implant were incubated overnight with FITC-conjugated anti-rat GFP (1:100, Sigma-Aldrich) and 7-amino actinomycin D (7-AAD, Apoptosis Detection Kit; BD Biosciences, Franklin Lakes, NJ) at 4 °C. Apoptotic cells were then counted and calculated as a percentage of the total cells.

### Osteogenic differentiation assay

MSCs were cultured in osteogenic culture medium containing 1.8 mM KH_2_PO_4_ and 10 nM dexamethasone (Sigma-Aldrich). After 28 days of osteogenic induction, an expression of the osteogenic marker runt-related transcription factor 2 (Runx2, 1:100, Santa Cruz Biotechnology) was determined by immunofluorescent staining.

### Adipogenic differentiation assay

MSCs were cultured in adipogenic culture medium containing 0.5 mM isobutylmethylxanthine, 60 μM indomethacin, 0.5 μM hydrocortisone, and 10 μg/ml insulin (all Sigma-Aldrich). After 14 days of adipogenic induction, expression of the adipogenic marker, peroxisome proliferator-activated receptor gamma (PPARγ, 1:100, Santa Cruz Biotechnology), was determined by immunofluorescent staining.

### Isolation of oral mucosa epithelial cells

Oral mucosa epithelial cell (OEC) cultures were performed based on a previous report [19]. Briefly, oral mucosa derived from 4-day-old Wistar rats was incubated with dispase (1 × 10^3^ IU/ml) in Mg^2+^ and Ca^2+^-free PBS for 12 h at 4 °C. The oral epithelium was then peeled from the connective tissue using tweezers. The epithelium was dispersed by pipetting ten times and seeded onto dishes or Ti plates placed on the bottom of dishes. OECs were cultured in defined keratinocyte serum-free medium (DK-SFM; Invitrogen, Grand Island, NY) and gentamicin (50 μg/ml) on plastic in a humidified atmosphere of 95% air and 5% CO_2_ at 37 °C.

### OEC coculture with MSCs

OECs were cocultured indirectly with MSCs using Transwell® insert as a separator between the two cell types. Briefly, OECs were cultured at a density of 5 × 10^5^ cells/mL on mirror-surfaced pure Ti plates [15 mm diameter by 1 mm thickness, 0.19 μm roughness (Ra), Japan Industrial Standards Class 1 (equivalent for ASTM Grade I)] (KS40, Kobelco, Kobe, Japan). Transwell inserts without MSCs in the upper chamber served as controls (Fig. 7a). As shown in Fig. 7c, the upper chamber contained either 5 × 10^2^, 5 × 10^3^, 5 × 10^4^, or 5 × 10^5^ MSCs (in 0.5 ml of culture medium), while the bottom chamber contained 5 × 10^4^ OECs (in 1.5 ml of medium) for the various assays described below.

### Cell adhesion assays

OEC adhesion assays were conducted according to previously published methods [16, 20]. Non-adherent or weakly attached cells were removed by shaking (3 × 5 min at 75 rpm) using a rotary shaker (NX-20, Nissin, Tokyo, Japan). Adherent cells were then counted and calculated as a percentage of the initial count, which was used to define adhesive strength of the cells.

### Scratch assays

Scratch assays were performed on Ti plates to model wounding using various numbers of MSCs in the upper Transwell chamber. The techniques were conducted as described previously [16, 20]. Briefly, confluent OEC monolayers were scratched with a cell scraper and cultured for 48 h. OECs at the edge of the wound were visualized immunofluorescently using antibodies against actin to observe cell migration, and then the migrating cells were counted on the wound area.

### Statistical analysis

Data are presented as means ± standard deviation (SD). One-way analysis of variance (ANOVA) with Fisher’s least significant difference test was performed. Significance was established when *p* < 0.05. Experiments were performed with triplicate samples and were repeated three or four times to verify reproducibility.

## Results and discussion

Because MSC treatment is being introduced more widely as a clinically available therapy, the method of administration must be considered to better mitigate risk. Although for a number of other factors also need consideration, including cell source, cell donor condition, cell population, and timing of MSC administration, this study only focused on comparison between systemic and local injection of MSCs into a rat oral implant model.

Sealing and defense at the PIE–implant interface are very important because dental implants in the oral mucosa are at high risk of inflammation. However, sealing between the PIE and implant is much weaker than that between the junctional epithelium (JE) and teeth [3], possibly owing to an inferiority of adhesion structures at the PIE-implant interface [15]. We therefore aimed to assess the influence of MSCs during implant treatment. Our previous report showed a positive effect of systemically injected MSCs for the improvement of peri-implant tissue sealing and acceleration of tissue healing [11].

### HRP penetration on implant surface

**Fig. 2:**
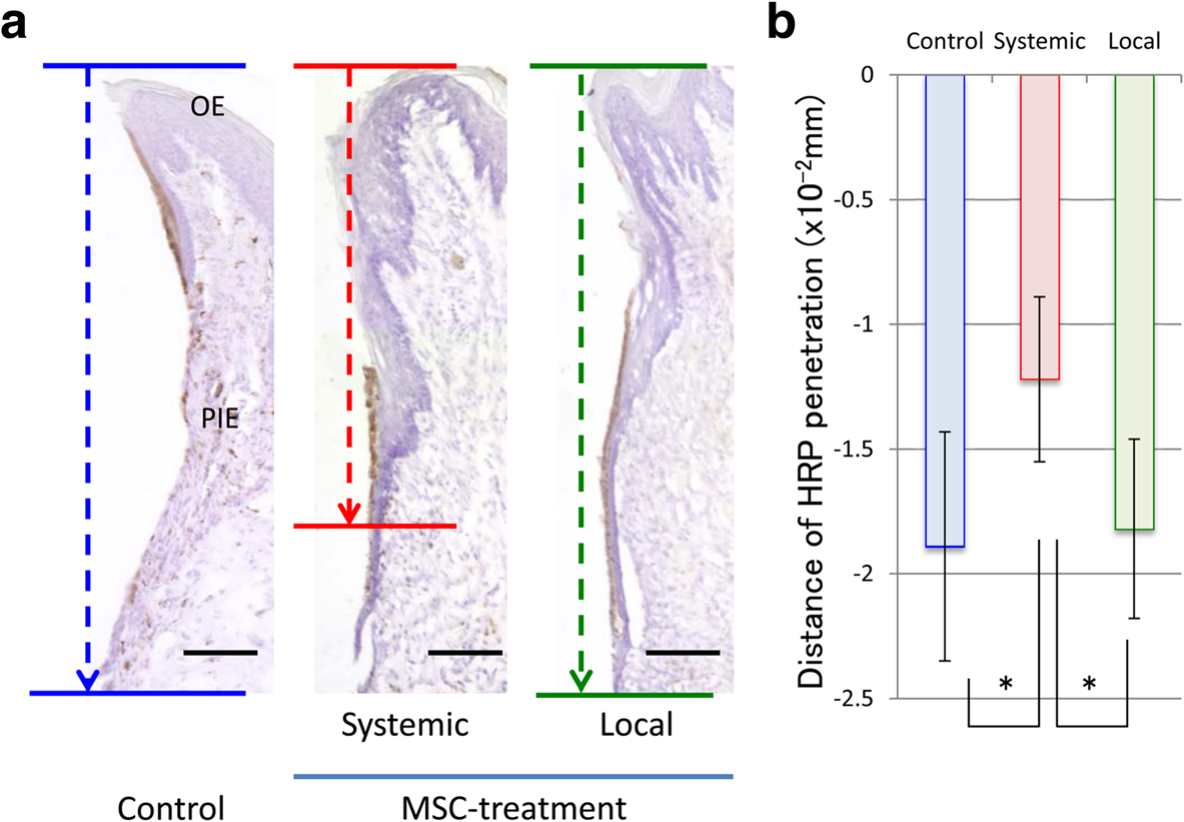
HRP penetration on implant surface**. a** Light micrographs of the epithelial structure around the control, local, and systemic group implants after HRP penetration. Bar = 200 μm. **b** Median distance of HRP penetration. Each bar represents the mean ± SD of the three independent experiments. *p < 0.05 vs. Cont

In the systemic group, a strong HRP reaction was seen only in the coronal portion of the PIE on the implant surface (Fig. 2a). In the control and local groups, HRP reaction was not only found in the coronal PIE region on the surface of the PIE but also in the connective tissue. Furthermore, in the middle and apical PIE regions of these latter groups, the deep layers of PIE cells exhibited the strongest HRP reaction. This result meant that the PIE with these groups had only a weak epithelial sealing, and had been penetration of the external factors to the surrounding tissue of implant.

The systemic group exhibited a significant improvement in blocking HRP penetration (Fig. 2b) compared with both the local and control groups, which were comparable.

### Distribution of Ln-332 in the peri-implant oral mucosa

**Fig. 3:**
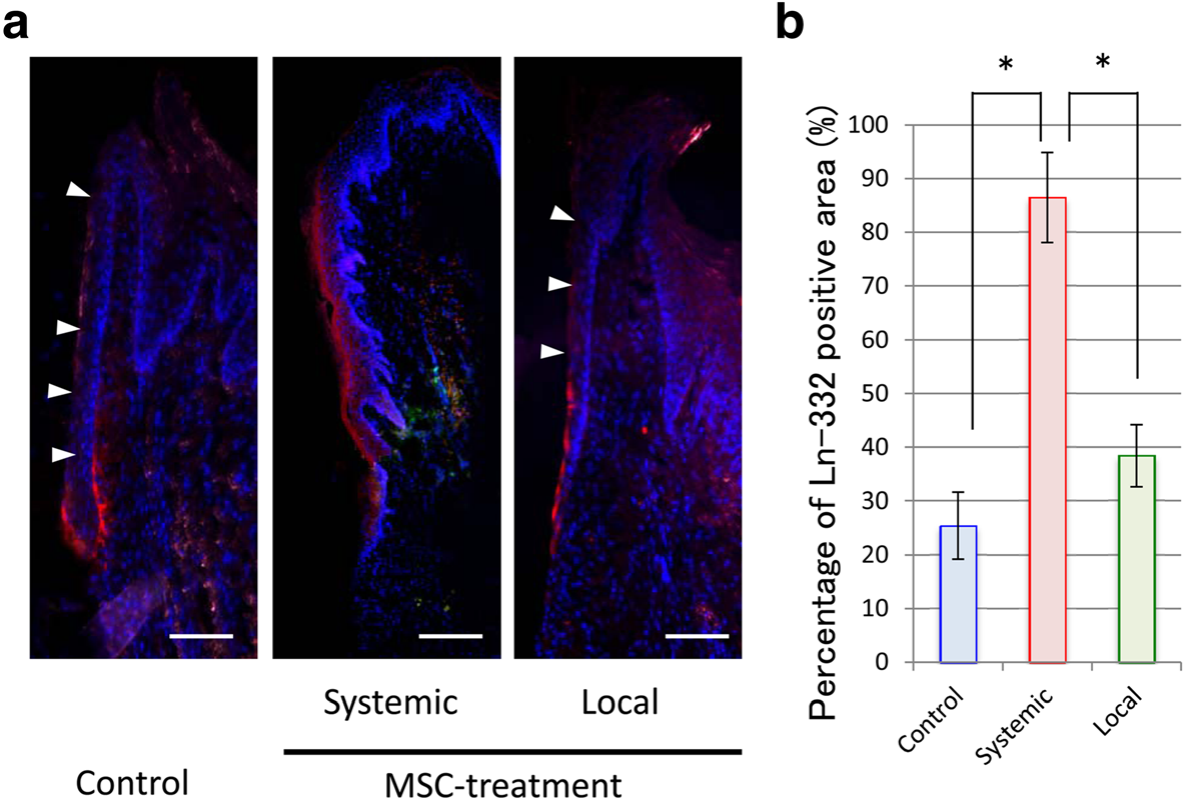
Ln-332 distribution on peri-implant epithelium (PIE) after MSC injection. **a** Light micrographs of Ln-332 distribution (red staining) in the gingiva around the control, local, and systemic group implants after 4 weeks. White arrowheads indicated lack of positive reaction. Blue staining: DAPI (bar = 200 μm) (**b**). Quantitation of Ln-332 presence in the PIE. Images were analyzed to quantify Ln-332 expression in the PIE around the implants. Each data point represents the mean ± SD of the three independent experiments. *p < 0.05

In the systemic group, immunohistochemical staining of Ln-332 showed a positive reaction along the whole implant-PIE interface at 4 weeks (Fig. 3a). In the local group, the Ln-332 deposition pattern in the PIE was comparable to that of the control group. In the oral mucosa around both local and control group implants, Ln-332-positive staining was apparent at the apical portion of the implant-PIE interface, but the upper portion of the interface did not exhibit Ln-332 detection. Only in the control group was the PIE-connective tissue interface intensely stained at the end of the PIE. Absence of Ln-332 staining was noted in the buccal mucosa underlying the OSE or OE in all groups.

As shown in Fig. 3b, expression of adhesion proteins on the interface between PIE-implant was significantly lower in the control and local groups compared with the systemic group.

Ln-332 is the major adhesive ligand for integrin α6β4, which interacts with the cytoskeletal elements, and is a component of the hemidesmosomes, epithelial adhesion plaques that tack the plasma membrane of the epithelial cells [21–23]. Moreover, Ln is expressed at the interface between the JE and natural tooth [24, 25] and is thought to be critical for the attachment of gingival epithelial cells to substrates [26, 27]. In our previous study, Ln was implicated in the adhesion of the PIE to the dental implant [20, 28]. Therefore, we observed the distribution of Ln during PIE formation around the implant to eliminate the influence of transplanted MSCs on the OE.

Connecting Ln and α6β4 integrin activates intracellular signaling pathways, such as the mitogen-activated protein kinase (MAPK) and phosphoinositide 3-kinase (PI3K) signaling pathways, which control cell migration, adhesion, and survival [29–31]. Our previous study showed that insulin-like growth factor-1 (IGF-1)-activated PI3K signaling promoted epithelial adhesion via HD activation of PI3K signaling and improved epithelial sealing around the implant [32]. Some studies indicate that MSCs activate intrinsic MSCs or various other cells through paracrine expression of IGF-1, epidermal growth factor (EGF), or platelet-derived growth factor (PDGF). Therefore, we highlight the importance of direct contact between MSCs and epithelial cells in order to change cell characteristics or activate cell differentiation.

### Whole body MSC accumulation

**Fig. 4:**
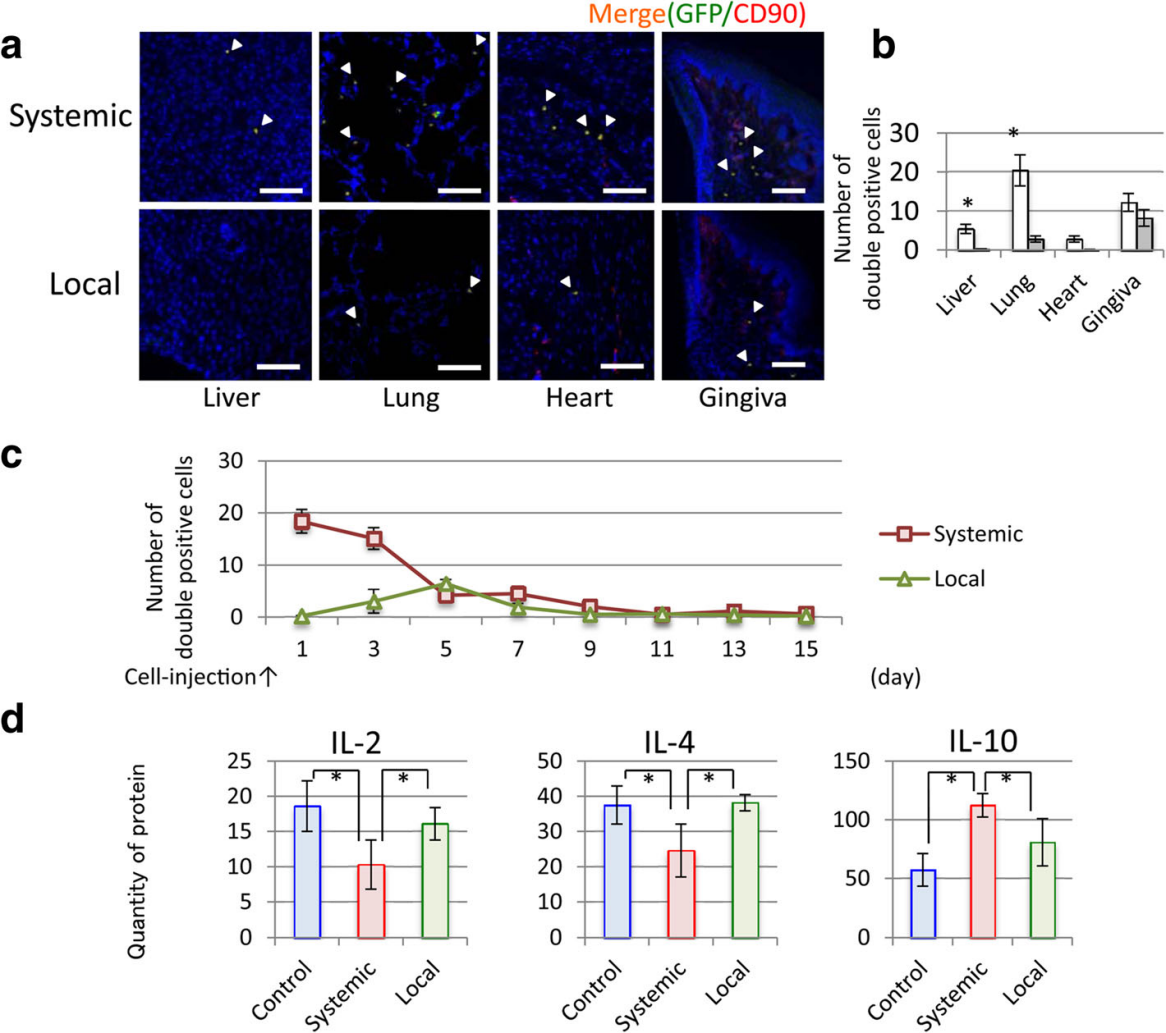
Whole body accumulation of MSCs. **a** CD-90/GFP-FITC double-positive cell (white arrowhead) visualization at day 1. (Bar = 200 μm) (**b**) CD-90/GFP-FITC double-positive cell counts in various organs in the control, systemic (white), and local (gray) groups at day 1. **c** Change of intravascular MSCs number up to 14 days after systemic or local MSC injection. **d** Levels of serum inflammatory cytokines, IL-2, IL-4, and IL-10 in control, local, and systemic groups

GFP/CD-90 double-positive cells were detected and counted in various tissues, including the mucosa around the experimental implants (Fig. 4a, b). Although few double-positive MSCs were observed in the liver and heart 1 day after MSC injection, double-positive MSCs were observed in the lung and peri-implant tissue after both systemic and local injection. Figure 4c shows changes in MSC numbers in the rat blood over time. In the local group, intravascular MSC number peaked at day 5, while in the systemic group, MSC number declined quickly at the early time points 3 days after the administration.

Subcutaneously administrated cells or drugs are reported to take a few days to be delivered into the body through vessel bloods [33, 34]. This may be owed to difficulty of the cells in securing vascular accesses to the target site because of a lack of blood vessels at the buccinators, while systemic MSC homing occurs more readily through the bloodstream [35].

The effects of MSC treatment on levels of serum inflammatory cytokines IL-2, IL-4, and IL-10 in the implant model rat are shown (Fig. 4d). Systemic MSC injection resulted in lower IL-2 and IL-4 levels and higher IL-10 levels compared with local MSC injection and the control.

### Accumulation of MSCs at the peri-implant tissue

An interesting study disclosed that intraperitoneal MSCs migrated and engrafted at the inflamed colon and passed through the whole intestinal wall reaching the luminal side [36]. Although we were unable to trace the exact migration of our locally administrated MSCs by observed fragmentary, in vivo imaging or tracking with superparamagnetic iron oxide might enable this using a series of flow [14, 37].

**Fig. 5:**
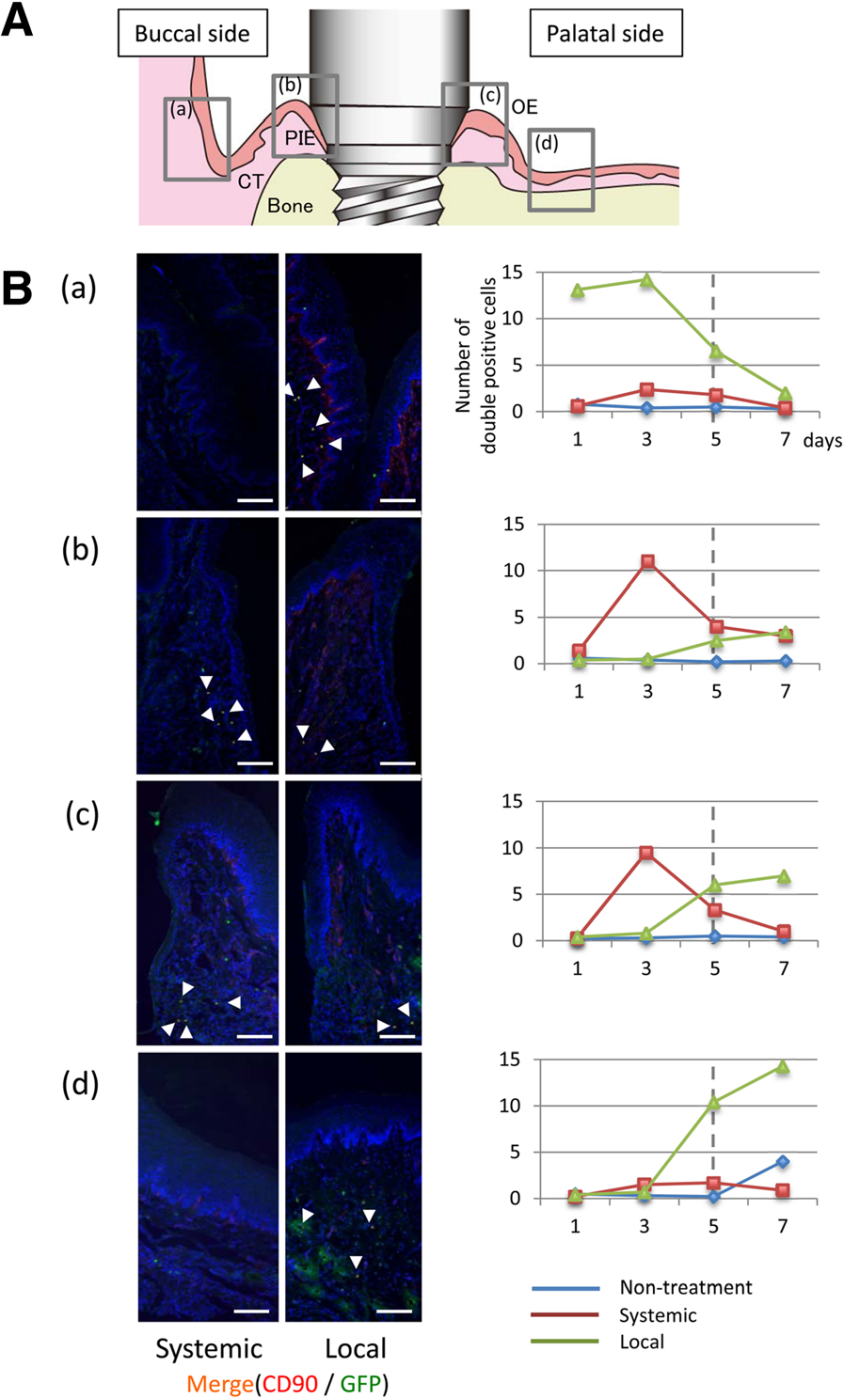
MSC accumulation in the peri-implant tissue. **A** Schematic of the tissue structural arrangement around the implant. Gray squares indicates panels (**B**, a) to (**B**, d). **B** Immunofluorescence photos around the experimental implants. Right panels show a graphical representation of the results. CD90 (red staining)/GFP-FITC (green) double-positive cells were detected by immunostaining 5 days after each administration. Data are means ± SD of four independent experiments. *p < 0.05 (bar = 200 μm). (a) Alveololingual sulcus around the point of local administration. (b) Buccal side of peri-implant mucosa. (c) Palatal side of peri-implant mucosa. (d) Palatal mucosa

In this study, GFP-MSCs took several days to be observed at the target organ after local injection (Fig. 5B (b, c)). Some cells were observed in the mass of the injected area (Fig. 5B(a)), while others were observed indirectly circulating within the whole body or were slightly accumulating at the wound area (Fig. 5B (d)). Specially, these results showed that the most of injected MSCs in the local group got delayed to accumulate around the implant. In the systemic group, GFP/CD-90 double-positive cells were observed around the apical portion of the PIE-like epithelial structure at days 3 and 5 (Fig. 5B (b, c)), after which positive staining declined over time. In the local group, MSC location was limited to the buccal mucosa near the experimental implant at early stage; however, MSC accumulation was observed at the mucosa around the implant from day 5 onwards (Fig 5B). On the contrary, the MSCs did not accumulate on the implant surface, unlike in the systemic group, and they remained around the implantation site for approximately 1 week.

### Detection of apoptotic GFP-MSCs

**Fig. 6:**
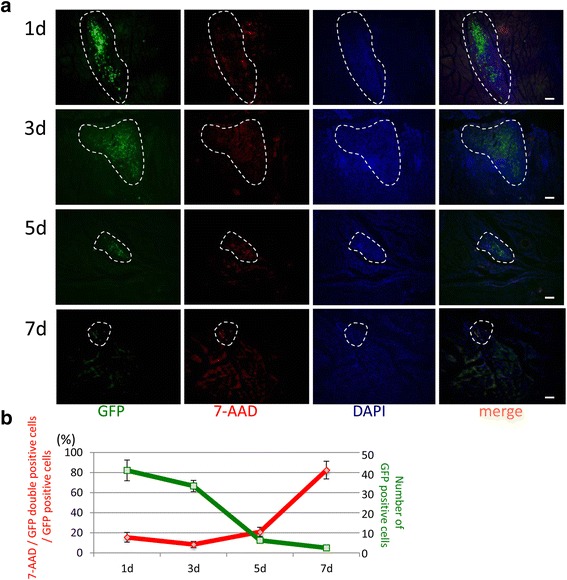
MSC apoptosis after local administration. **a** GFP-conjugated MSCs (green) were stained by 7-AAD (red) as an apoptosis marker and nuclear DAPI (blue). (Bar = 80 μm) **b** Quantitation of 7-AAD/GFP double-positive or GFP-positive cell numbers in the mass (bound area by white dotted line) after local MSC administration

Due to the existence of muscles, connective tissue, dermal layer, and basement membrane, cells within the mass of the injected area encounter these barriers, inhibiting the distance of migration between the application region and inflammatory site, which has an estimated diameter of 20–30 μm (Fig. 6). High-density cell injection at the topical region is also an obstacle for homing, thus using a vasodilator like heparin, culturing the cells under hypoxic condition, maintaining a lower confluence, or the addition of IL-3, IL-6, IGF-1, tumor necrosis factor alpha (TNF-α), or interferon-gamma (IFN-γ), can be used to increase C-X-C chemokine receptor type 4 (CXCR-4) [38], which is a specific receptor for stromal-derived-factor-1 (SDF-1, also called CXCL12), an MSC chemotactic factor that could improve homing efficiency [39]. 

Locally injected MSCs decreased sharply in number from the buccal site within a week (Fig. 6a). One day after injection, some 7-AAD-positive cells were detected within the MSC mass at the subcutaneous tissue. From days 3 and 5, MSCs began to undergo apoptosis within the local administration region. At day 7 after injection, the level of MSC apoptosis was approximately 90% (Fig. 6b).

### Relationship between cell density and differentiation

**Fig. 7:**
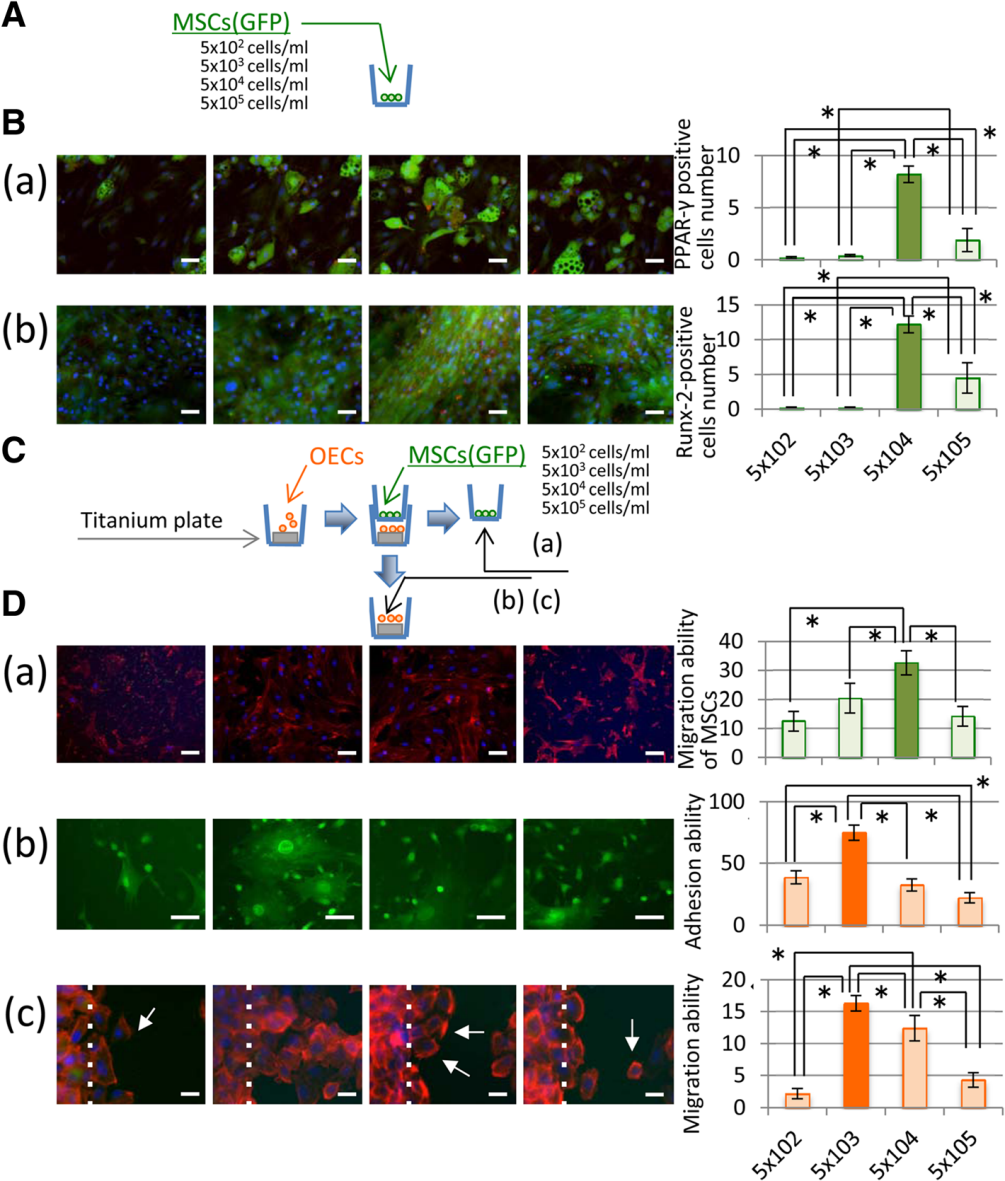
Relationship between cell density and differentiation. **A** MSCs were seeded at four concentrations (5 × 10^2^, 5 × 10^3^, 5 × 10^4^, 5 × 10^5^ MSCs/ml) into culture wells. **B** Multipotent differentiation of MSCs related to cell density. (a) Adipogenic differentiation of MSCs. The graph shows quantification of PPAR-γ-positive cell numbers as differentiated adipocytes from independent experiments (means ± SD). (Bar = 40 μm) (b) osteogenic differentiation of MSCs. The graph shows quantification of Runx-2-positive cell numbers as differentiated osteoblasts from four independent experiments (means ± SD). (Bar = 50 μm) **C** schematic of in vitro coculture study. **D** Relationship between MSCs and OECs in coculture. (a) Quantification of MSCs number in the upper Transwell insert chamber to determine MSC migration through the 8-μm pores. (Bar = 20 μm) (b) OEC adhesion assay. Data show the percentage of OECs under MSC coculture. Bars represent the means ± SD of four independent experiments. *p < 0.05 (c) scratch assay. OECs were cultured for 3 days then observed by immunofluorescence staining for actin filaments (red). The white dotted line shows the limit of the wound area into which the cells migrated, while the white arrow indicates migrating cells. (Bar = 20 μm) the right panel graphically shows the number of migrating cells under each condition. Bars represent the means ± SD of four parallel experiments. *p < 0.05

Isolated MSCs were seeded at four concentrations (5 × 10^2^, 5 × 10^3^, 5 × 10^4^, 5 × 10^5^ MSCs/ml) in adipogenic and osteogenic induction conditions. When the medium was switched to adipogenic and osteogenic differentiation medium, the MSC concentrations were approximately 30, 60, 80, and 100%, respectively. MSCs differentiation into adipocytes and osteoblasts was determined by PPARγ and Runx2 immunofluorescence staining, respectively (Fig. 7 A, B). At a density of 5 × 10^4^ MSCs/ml, adipogenic and osteogenic differentiation were confirmed by increased expression of specific adipogenic markers and Runx2, respectively, using western blotting. Therefore, cells at a suitable cell density within the MSC mass were induced to undergo osteogenic or adipogenic differentiation. However, 3 days after injection, the MSCs began to undergo apoptosis over time in the local administration region. A high ratio of apoptosis occurred immediately after local administration of MSCs, which reduced the amount of viable cells at early stage.

### MSC migration from the local mass

Figure 7C and D showed the suitable amount of MSCs had much better positive effect for the migration and adhesion of OECs to titanium surface. MSC attachment within the upper Transwell chamber was determined 24 h after seeding by fluorescence microscopy, as shown in Fig. 7D (a). The majority of MSCs appeared flattened with numerous cytoplasmic extensions and lamellipodia. The majority of MSCs passed through the transwell pores when seeded at a density of 5×104. However, at a density of 5 × 104 the MSCs more readily passed through the 8 µm pores compared with cells at other densities.

### OEC and MSC coculture at various seeding densities.

MSCs were seeded at a range of densities (5 × 10^2^, 5 × 10^3^, 5 × 10^4^, 5 × 10^5^ cells/ml) within the upper Transwell chamber, and OECs were cultured on titanium plates like as titanium implant surface, as shown in Fig. 7D (b, c). Only at a density of 5 × 10^3^ did the MSCs activate OEC migration and adhesion.

Furthermore, ELISA indicated that this may not have regulated inflammation because there was no significant difference in expression of IL-10 detected at 24-hour post injection compared with the control (data not shown). The blood stream is thought to be a suitable environment for MSCs, since their survival is higher in this source than within inflamed tissues [37]. To therefore ensure a constant number of viable cells, repeat doses with smaller cell numbers or scatter injection points may benefit local MSC administration. This may permit more cells to be intravasated into the blood vessels, and offer an antiphlogistic effect to the inflammation sites. In terms of MSC administration timing, an earlier response is believed to be more effective to clinical outcomes [36, 40], although similar results have been obtained following delayed administration in some studies. Above all, investing research efforts in identifying the most efficacious route for MSC delivery is a critical matter because there is currently no consensus.

## Conclusions

Our study supports systemic administration of MSCs to enable accelerated soft tissue sealing of the Ti surface in our rat oral implantation model. Although local MSC administration had little positive effect in our model, the MSCs accumulated around the peri-implant oral mucosa and were identified in various organs, indicating a wide range of possible applications. This study highlights that clinical cases should be considered individually prior to practical application of MSCs, and that further investigations are needed to understand the exact cellular and molecular mechanisms of MSCs following their local administration.
